# Zinc deficiency induces abnormal development of the myocardium by promoting SENP5 overexpression

**DOI:** 10.1371/journal.pone.0242606

**Published:** 2020-11-19

**Authors:** Xiaoyu Zhang, Cuancuan Wang, Dan Zhao, Xuhong Chen, Chunyan Zhang, Jun Zheng, Xiaozhi Liu

**Affiliations:** 1 Department of Neonatology, Tianjin Medical University, Tianjin, P.R. China; 2 Department of Cardiology, Tianjin Fifth Central Hospital, Tianjin, P.R. China; 3 Department of Neonatology, The Second Hospital of Tianjin Medical University, Tianjin, P.R. China; 4 Department of Obstetrics and Gynecology, Tianjin Fifth Central Hospital, Tianjin, P.R. China; 5 Department of Pharmacy, Tianjin Binhai New Area Hospital of Traditional Chinese Medicine, Tianjin, P.R. China; 6 Department of Neonatology, Tianjin Central Hospital of Gynecology Obstetrics, Tianjin, P.R. China; 7 Central Laboratory, The Fifth Central Hospital of Tianjin, Tianjin, P.R. China; Texas A&M University College Station, UNITED STATES

## Abstract

Gestational zinc deficiency is a cause of congenital heart disease in the fetus, and sentrin/small ubiquitin-like modifier (SUMO)-specific proteases (SENPs) as deSUMOylation enzymes play a crucial role in the development of cardiac structures. However, current studies of the regulation and function of SENP in zinc-deficient status during heart development remain limited. In this study, SUMO1 modification was found to gradually decrease during heart development, and the level of SENP5 exhibited a similar trend to SUMO1 conjugation. In addition, zinc deficiency resulted in cardiac dysplasia, increased cell apoptosis, decreased cell viability, and differentiation inhibition of hiPSC-CMs. In order to investigate the function of SENP5 in zinc deficiency, hiPSC-CMs were transfected with SENP5 small interfering RNA. The negative effects of zinc lacking conditions were reversed with depletion of SENP5. It was confirmed that zinc deficiency induced abnormal differentiation of hiPSCs and increased apoptosis of hiPSC-CMs by promoting SENP5 overexpression, which led to cardiac dysplasia. Thus, it was concluded that SENP5 regulates the SUMO1 deconjugation during heart development and zinc deficiency may reduce conjugated SUMO by promoting SENP5 overexpression, which induces abnormal development of the myocardium.

## Introduction

Congenital heart disease (CHD) is a common congenital malformation that endangers the life and health of newborn babies and accounts for the highest incidence of birth defects in infants [1]. Furthermore, the etiology is extremely complicated. It is believed that genetic and environmental factors are involved in the occurrence of CHD. Heart development is a sophisticated process controlled by multiple transcription factor families, such as myocyte enhancer factor-2 (MEF2), GATA, Tbx, Nkx2, and Hand, which connect gene and signaling pathways to regulate myocardial gene expression, muscle and vessel growth, and morphogenesis [2]. Additionally, various molecular disturbances in the factors regulating the specification and differentiation potential of cardiomyocytes, such as mechanical forces, BRG1-SWI/SNF, and SDF1/CXCR4 [3–5], can lead to congenital heart disease.

Zinc is an important microelement in cells, which participates in the synthesis and activity regulation of various metalloenzymes such as transcription factors, alcohol dehydrogenase, copper-zinc superoxide dismutase, carbonic anhydrase, and RNA polymerase. Therefore, zinc is involved in regulating gene transcription, protein translation, and maintaining the stability of organelle structures [6]. In the USA, the physiological demand for zinc in pregnant women has increased, and an additional 2–4 mg zinc per day is recommended compared with non-pregnant women [7]. Recent studies have shown that zinc deficiency during pregnancy can cause malformations during embryonic heart development [8]. In rats, severe maternal Zn deficiency has been associated with an increased risk of fetal heart anomalies that were speculated to result in the distribution of Cx43 and HNK-1 in fetal hearts [9]. Duffy *et al*. established a model of zinc deficiency by feeding rats a Zn-deficient diet during pregnancy and found that zinc deficiency caused abnormal changes in the morphology of cardiomyocytes, such as an absent innominate artery, thin ventricular wall, and ventricular septal defects [10]. Additionally, the Zn metalloenzyme Adamts plays an important role in myocardial development [11–13]. However, the adverse effects of zinc deficiency on fetal heart development and their mechanisms have not been elucidated.

SUMOylation is a dynamic, reversible post-translational modification that alters the conformation, localization, and stability of the target protein, and is involved in regulation of the cell cycle, gene transcription, chromatin remodeling, stem cell maintenance and differentiation, DNA damage repair, and ribose biosynthesis [14]. In the last decade, SUMOylation had has been reported to play an essential role in regulating and controlling cellular functions, including differentiation and developmental processes [15, 16]. SENPs play an important role in maintaining the dynamic balance of SUMO modification and ensuring normal physiological functions of cells and organisms [17]. Studies have confirmed that SENPs play an important role in embryonic development [18, 19] and that knocking out SUMO2 or SENP2 causes embryonic lethality [20–22]. Furthermore, several cardiac development-related transcription factors have been reported to be involved in cardiac development and differentiation of cardiomyocytes by conjugation of SUMO with targets such as GATA4, Nkx2.5, and Islet-1 [23, 24]. Delineating these mechanisms will become the basis to understand the dynamics of specific congenital heart diseases and develop therapies for fetuses, postnatal cardiac defects and heart failure.

Therefore, we examined the spatiotemporal changes of SUMO1 and SENPs during development of the heart and explored the association of SENPs and zinc deficiency induces abnormal development of the myocardium.

## Materials and methods

### Animal model and experimental design

Mouse experiments were performed according to the regulations and guidelines approved by the Animal Ethics Committee of the Fifth Central Hospital of Tianjin (Tianjin, China, TJWZX2018064). Animal studies were adhered to the Guide for the Care and Use of Laboratory Animals (8th edition, 2011) and the guidelines for animal research reporting in vivo experiments (ARRIVE) (Supplemental Digital Content 1, Checklist, https://www.nc3rs.org.uk/arrive-guidelines). Two female and one male mice in each cage were fed under controlled temperature between 22 and 26°C and stable humidity between 40% and 60% in a 12-hour light/dark cycle and provided with sufficient food and water. The mice were checked twice a day for a vaginal plug, and the day of finding a plug was defined as embryonic day (E) 0.5. Pregnant female mice were provided with a zinc-deficient diet (2.78 mg/kg zinc; ZnD) as the zinc deficiency group or a standard diet (29.83 mg/kg zinc; ZnA) as the control group from first day of pregnancy. The pregnant mice were anesthetized with sodium pentobarbital (50mg/kg, i.p.), then fetuses were removed by cesarean section at E10.5–E19.5 and sacrificed by decapitation to acquire heart tissue. At the end of the experiments, the mice were executed by cervical dislocation. Neonatal mice were sacrificed via cervical dislocation, followed by a sternotomy. Lungs were collected and embedded for further study at postnatal day 1 to 7 and 14 (P1 to P14).

### Culture of hiPSCs and induction of hiPSC-CMs

Newly generated hiPSCs were cultured under defined xeno-free culture conditions on PSCease hiPSC basement membrane matrix coated to culture plates in PSCease hiPSC complete medium (0.7 mL additives mixed with 50 mL basal medium), which was changed daily during the procedure, and directly covered with sterile coverslips. The cells were incubated at 37°C with 5% CO_2_ and subcultured every 3–4 days with 0.25% tryptase and 0.02% EDTA. The EDTA solution was removed and PSCeasy iPSC medium (2 ml/well) was added to the cells, followed by dispersion of the cells. For cardiomyocyte induction, hiPSCs were subcultured in vitronectin-coated 6-well plates and then replated on vitronectin in PSCeasy iPSC complete medium and cultured until 80%–90% confluence. The cells were then cultured in CadioEasy human cardiomyocyte differentiation medium I with the medium changed to CadioEasy human cardiomyocyte differentiation medium II after 2 days. Then, at 4 days, the medium was exchanged with CadioEasy human cardiomyocyte differentiation medium III that was changed every 2 days. Close observation of cell growth, recording of obvious cell morphology changes, and fluctuations in cardiomyocytes were performed daily to confirm induction of hiPSC-CMs. To investigate the effects of zinc deficiency, TPEN [N,N,N′,N′-tetrakis-(2-pyridylmethyl) ethylenediamine] was added to the medium at 10 μM for 2 h. TPEN is an amino chelator of transition metals, which induces zinc depletion. At 80%–90% confluence, each group of cells was transfected with a SENP5 small interfering RNA (siSENP5) that specifically knocked down the SENP5 gene and inhibited the expression of SENP5 in vivo and *in vitro*. The cells were divided into control, TPEN, control+siSENP5, and TPEN+siSENP5 groups.

### RNA interference

Small interfering RNA (siRNA) oligonucleotides were synthesized by Sigma (Sigma, Inc, USA). The sequences were as follows: negative control siRNA: 5′-UUCUCCGACCGUGUC ACGUTT-3′; SENP5 siRNA: SENP5-1, 5'-TGCTGTTGACAGTGAGCGACCAGTTTACTTGGAATAGACAGTAGTGAA GCCACAGATGTA-3'; SENP5-2, 5′-GCAGGTGAAGCATTTCCAGTGTAATA AGG-3′; SENP5-3, 5′-CTGGAGCAGACAA ATCTGTGAGTAGTGTA-3′. SiRNAs were transfected into cells using Lipofectamine 3000 (Invitrogen, Carlsbad, CA), according to the manufacturer’s protocol. Cells were incubated at 37°C for 48 h.

### Immunofluorescence

HiPSC-CMs were fixed with 4% paraformaldehy for 30 min at room temperature. Then, 0.2% Triton X-100 was added at room temperature for 10 min, followed by 10% sheep serum at room temperature for 30 min. The sheep serum was rinsed off gently, and cells were incubated with a primary antibody (anti-cTnT, 1:200, ab8295; Abcam, Cambridge, MA, and anti-cTnI, 1:200, ab47003; Abcam) diluted with PBS in an appropriate proportion at 4°C overnight. The cells were washed with PBST three times and then incubated with the secondary antibody diluted in PBS for 1 h at 37°C. Images were obtained under a fluorescence microscope.

### Cell counting kit-8 (CCK8) assay

A cardiomyocyte suspension was seeded into a 96-well plate at 2,500 cells/well and incubated at 37°C with 5% CO_2_ and saturated humidity. After 24 h, the medium was discarded and various concentrations of TPEN were added for 30 min at 37°C. Then, 10 μL of CCK8 working solution was added to each well and the cells were incubated for a further 2 h. The OD value was measured at 450 nm with a microplate reader.

### Flow cytometry

Cells were collected (1–5×10^6^ cells/mL), centrifuged at 800 *g* for 5 min, washed once with PBS, and then fixed in cold 4% paraformaldehyde at 4°C for 1–2 h. After centrifugation, the supernatant was discarded and the cell pellet was resuspended in PBS for 5 min. The cells were filtered once through a 400-μm mesh, centrifuged at 800 *g* for 5 min, and the PBS was discarded. Annexin V and PI were added in the dark for 30 min at 4°C. The excitation wavelength was 488 nm and the emission wavelength was >630 nm.

### Western blotting

Equal amounts of proteins were electrophoresed on SDS-PAGE gels and then transferred to a PVDF membrane. Membranes were probed overnight at 4°C with the following primary antibodies (all from Abcam): Sox2 (1:1000, ab97959), cTnI (1:2000, ab47003), SENP1 (1:1000, ab108981), SENP2 (1:1000, ab131637), SENP3 (1:1000, ab71677), SENP5 (1:1000, ab58420), SENP6 (1:1000, ab77619), SENP7 (1:200, ab224752), GAPDH (1:1000, ab8245), Bcl-2 (1:1000, ab692), and caspase-3 (1:1000, ab2302). Primary antibody binding was detected by secondary antibodies and visualized by the enhanced chemiluminescence method.

### Histology and immunohistochemical analysis

Complete and clear paraffin-embedded heart sections were selected. Sections were dewaxed with xylene and treated with an alcohol gradient. Next, the paraffin-embedded sections were placed in 0.01 M citrate buffer and autoclaved for antigen retrieval. An endogenous peroxidase blocker (reagent A) was added dropwise, followed by incubation at room temperature for 10 min in the dark to inactivate endogenous peroxidase activity. Goat serum was added dropwise and allowed to stand at room temperature for 30 min. Then, the primary antibody was added dropwise, followed by overnight incubation at 4°C. Primary antibodies (all from Abcam) against SENP1 (1:200; ab108981), SENP2 (1:200; ab58418), SENP3 (1:100; ab71677), SENP5 (1:200; ab58420), and SENP7 (1:200; ab224752) were used. The sections were then incubated with horseradish peroxidase-labeled polymer (reagent B) for 30 min at 37°C. DAB color development was applied for 3–5 min, and the sections were observed under a microscope until the section showed a caramel color. Hematoxylin staining of the nucleus was conducted for 4 min, followed by treatment with 1% hydrochloric acid alcohol, saturated lithium carbonate, alcohol, and xylene in that order. Finally, a suitable amount of neutral resin was applied on the sections that were then covered with a coverslip. Photographs were obtained under the OLYMPUS microscope.

### Statistical analysis

Statistical analysis was performed as the mean ± SD using GraphPad Prism 6.0 software (GraphPad Software, Inc.). All experimental data were randomly assigned to different experimental groups (n≥3). Significant differences were analyzed by one-way ANOVA followed by Tukey’s test. *P* < 0.05 was considered as statistically significant.

## Results

### Spatiotemporal distribution characteristics of SUMO1 during fetal mouse development

**Fig 1:**
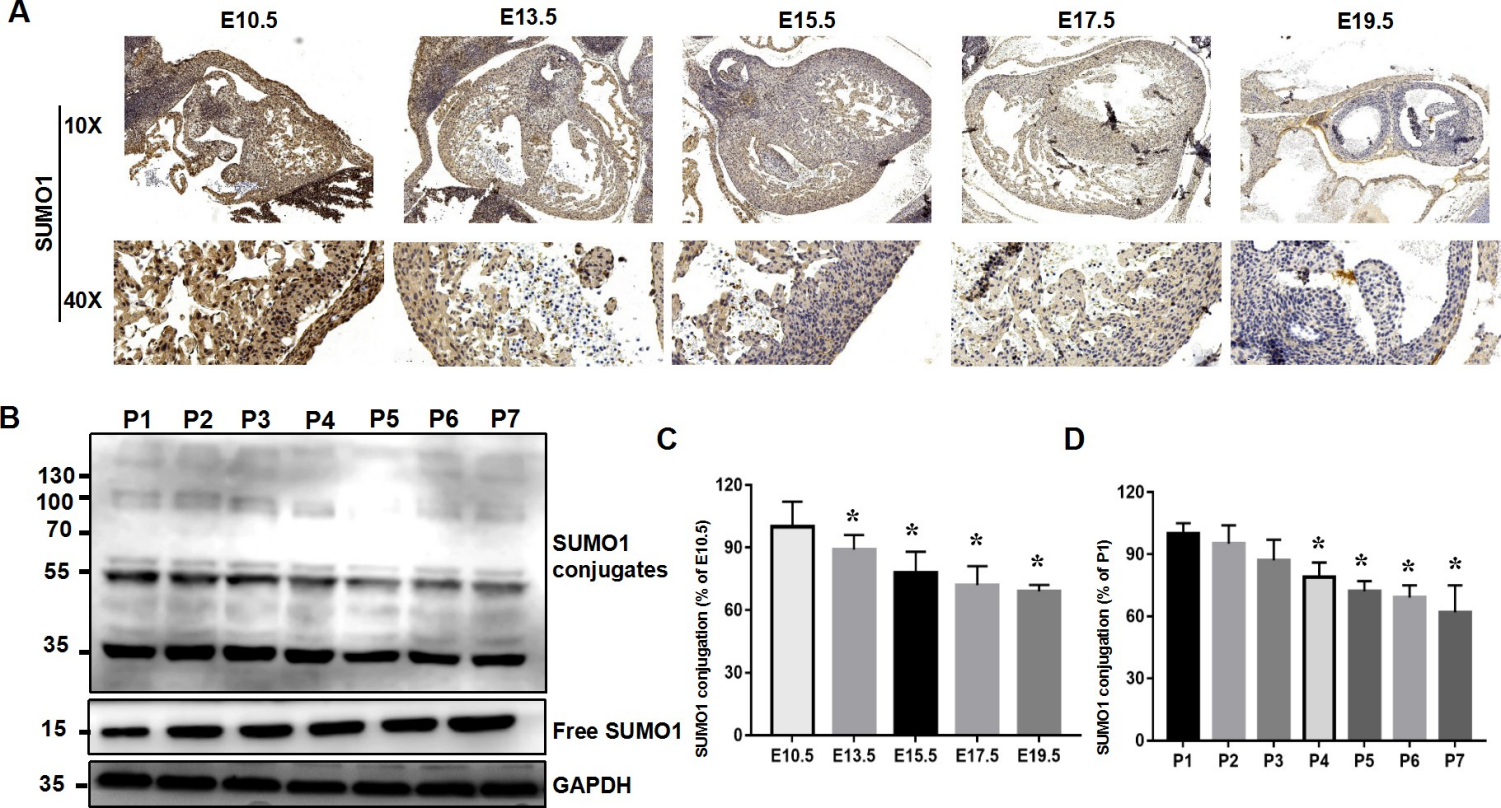
Spatiotemporal distribution characteristics of SUMO1-modified proteins during fetal mouse development. (A) Immunohistochemistry of SUMO1-modified proteins from E10.5 to E19.5 (n = 4). (B) Western blotting of SUMO1-modified proteins and free SUMO1 from P1 to P7 (n = 6). (C) Quantitative analysis of SUMO1-modified proteins in (A) by ImageJ software. Data are presented as the mean ± SD. A significant change was observed at E10.5. * P < 0.05 vs. E10.5. (D) Quantitative analysis of conjugated SUMO1 protein expression in (B). Decreases of conjugated SUMO1 proteins were quantified at P1–P7. Statistical significance of conjugated SUMO1 protein at P7 was analyzed by Tukey’s test. Values are presented as the mean ± SD.* P < 0.05 vs. P1.

To explore the role of SUMOylation in heart development, E10.5–19.5 mouse embryonic heart specimens were collected and the level of SUMO1 conjugation was determined by IHC. The results showed that the level of SUMO1-modified protein was decreased during embryonic cardiac development (Fig 1A). The difference was statistically significant (*P*<0.05; Fig 1C). The mouse heart maintains a certain differentiation ability within 7 days after birth, which then disappears [25, 26]. Western blot analysis was performed to detect the variable level of SUMO1 conjugation from neonatal mice at postnatal day (P) 1–7. As shown in Fig 1B and 1D, free SUMO1 and a higher migrating band indicating SUMO1-conjugating proteins were observed. Quantitative analysis of conjugating SUMO1 proteins decreased gradually from P1 to P7 (P<0.05), while free SUMO1 exhibited a visible, progressive increase. The results indicate that the SUMOylation and deSUMOylation were maintained in a dynamic balance, and the levels of SUMOylated proteins were gradually decreased with the maturation of myocardium and cardiac growth.

### Spatiotemporal distribution characteristics of SENP family members during the development of newborn mice

**Fig 2:**
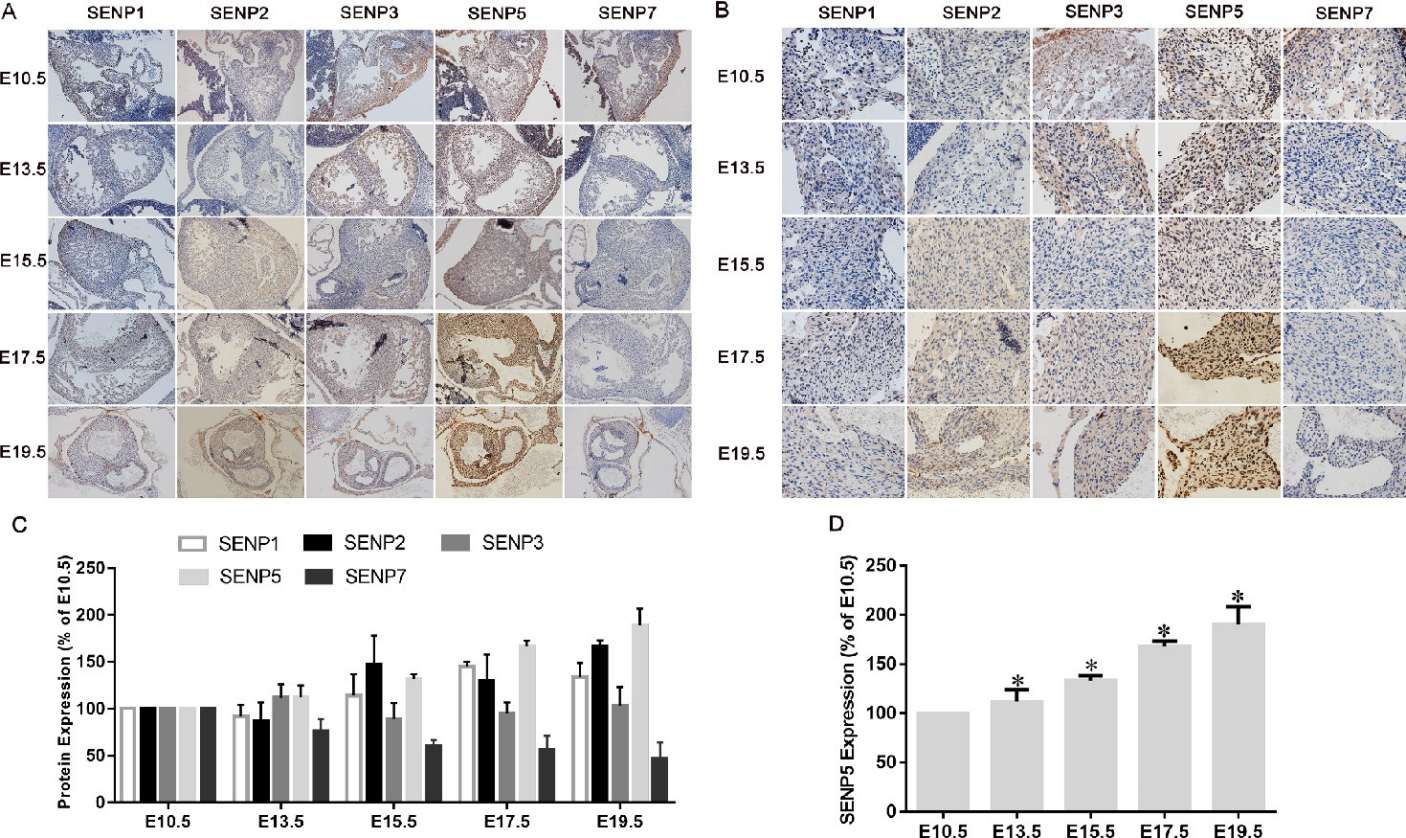
Spatiotemporal distribution characteristics of SENP family members during fetal mouse development. (A) Immunohistochemistry of SENP families expression from E10.5 to E19.5 (n = 4). (B) Enlargements of (A) (n = 4). (C) Relative levels of SENPs expression were quantified from E10.5 to E19.5. (D) Expression levels of SENP5. Statistical significance was analyzed using the t-test. Values are presented as the mean ± SD. Statistical significance was analyzed by the Tukey’s test. *P < 0.05 vs. E10.5.

**Fig 3:**
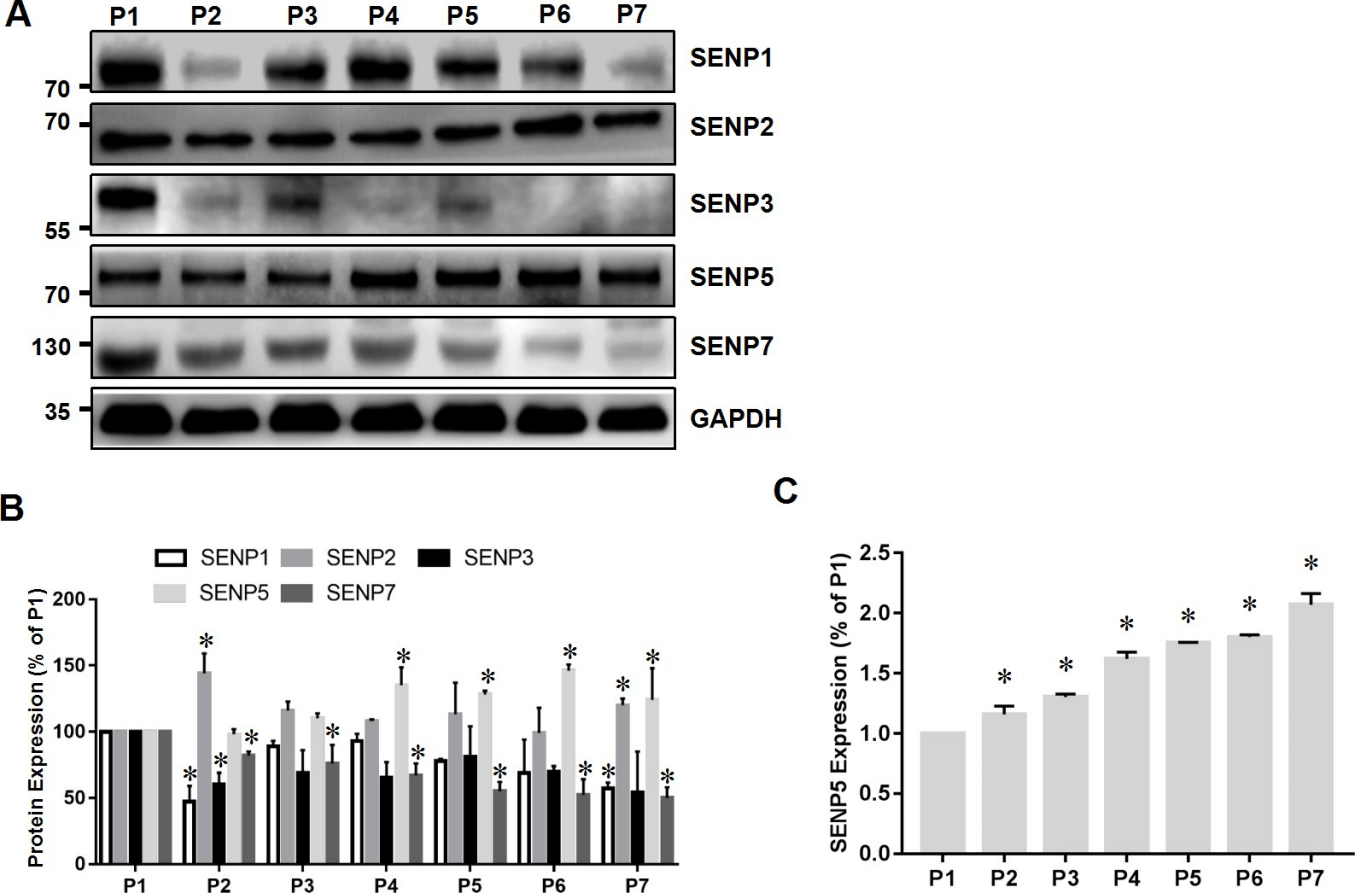
Spatiotemporal distribution characteristics of SENP family members during development of newborn mice. (A) Western blotting of SENP1, SENP2, SENP3, SENP5, and SENP7 protein expression from P1 to P7 (n = 6). (B) Quantitative analysis of SENP protein expression in (A). Values are presented as the mean ± SD. Statistical significance was analyzed using Tukey’s test. *P < 0.05 vs. P1. (C) Expression of SENP5 protein from P1 to P7. Values are presented as the mean ± SD. Statistical significance was analyzed using Tukey’s test. *P < 0.05 vs. P1.

To determine which de-SUMOylation enzyme was closely related with myocardial SUMO1-conjugated proteins, the role of SENP family members in heart development was explored by IHC. SENP1 protein decreased at E13.5, increased at E15.5 to E19.5, and finally presented a highest level at E17.5. Compared with E10.5, the expression level of SENP2 was decreased at E13.5 and increased at E15.5 to E19.5, while the expression level of SENP3 was adverse. The expression level of SENP7 protein was decreased, and SENP5 proteins were increased during heart development (Fig 2A and 2B). Next, we used ImageJ to analyze and GraphPad Prism 6.0 software to quantify the immunohistochemistry results (Fig 2C and 2D). In the mice embryo at E10.5–E19.5, compared with other SENP family members, expression of SENP5 protein was increased significantly, and gradually increased through the entire cardiac growth process (*P*<0.05). Furthermore, we collected cardiac specimens from neonatal mice at P1–P7 developmental stages and determined the expression of SENPs in the heart by western blotting. SENP5 was expressed highly at P1 and then increased gradually. Compared with other members of the SENP family, expression of SENP5 was decreased significantly (*P*<0.05; Fig 3A–3C). In addition, the protein level of SENP5 exhibited a relative trend with SUMO1 conjugation. These results further confirm that SENP5 may take an important part in heart development.

### Zinc deficiency induces a increasing level of SENP5

**Fig 4:**
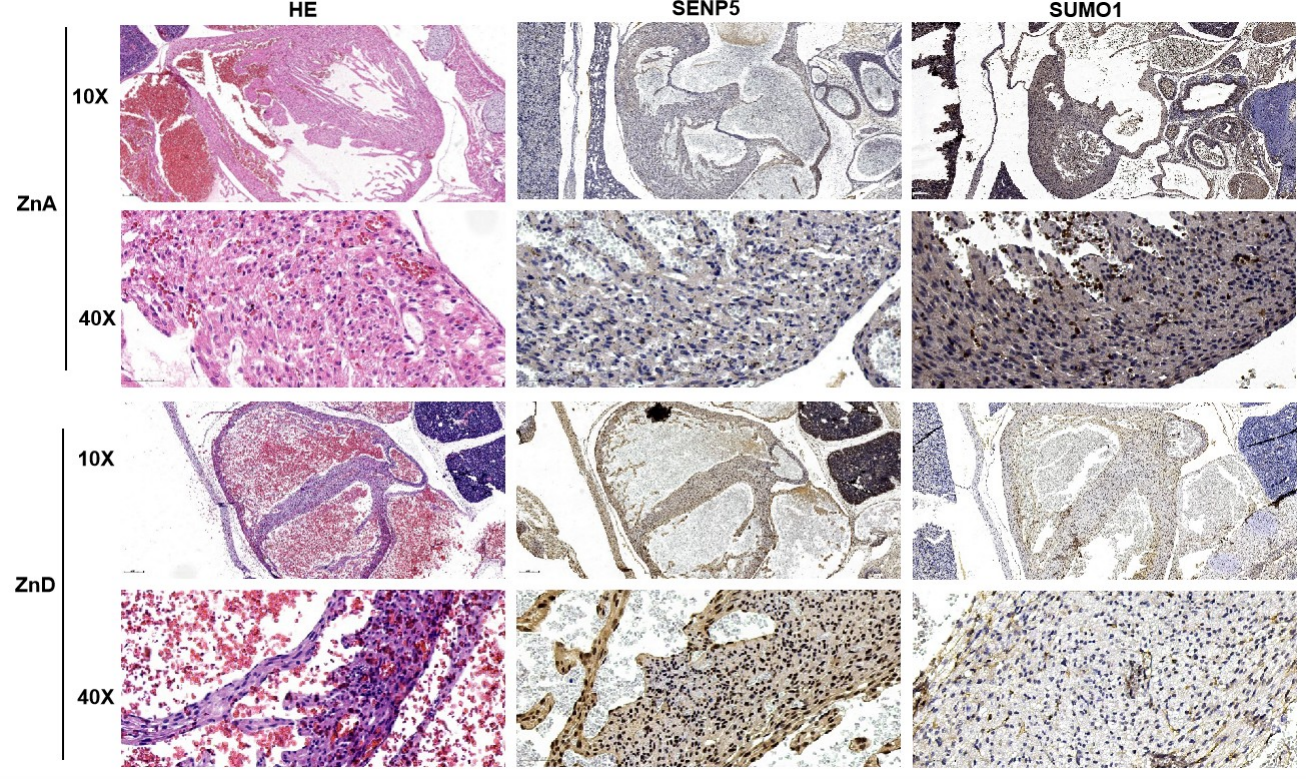
Difference in development of the myocardium and characteristics of SENP5 and SUMO1-modified proteins between zinc deficiency and control groups during fetal mouse development. HE staining and immunohistochemistry of SENP5 and SUMO1-modified proteins at E15.5 in zinc-deficient and control groups are shown (n = 3).

From day 1 of pregnancy until delivery, maternal mice were provided a zinc-deficient diet (2.78 mg/kg zinc; ZnD) as the zinc deficiency group or a standard diet (29.83 mg/kg zinc; ZnA) as the control group. Expression of SENP5 protein decreases significantly in the mouse embryo at E10.5–E19.5 stage [27]. Therefore, embryonic heart tissue was obtained from the two different groups to explore the spatiotemporal distribution characteristics of the SUMOylation and SENP5 protein by IHC. Compared with the ZnA group, development of the mouse embryonic heart was delayed, the thicknesses of both the ventricular wall and interventricular septum were decreased. Meanwhile, we observed the histologic abnormality of myocardium in the zinc-deficient group. The results of IHC revealed the expression of SENP5 was increased, whereas the SUMO1 modified proteins was decreased with deficiency of zinc (Fig 4).These results imply that zinc deficiency presented cardiac dysplasia, which maybe associated with overexpression of SENP5 protein.

### Zinc deficiency affects apoptosis, viability and differentiation of hiPSC-CMs *in vitro*

**Fig 5:**
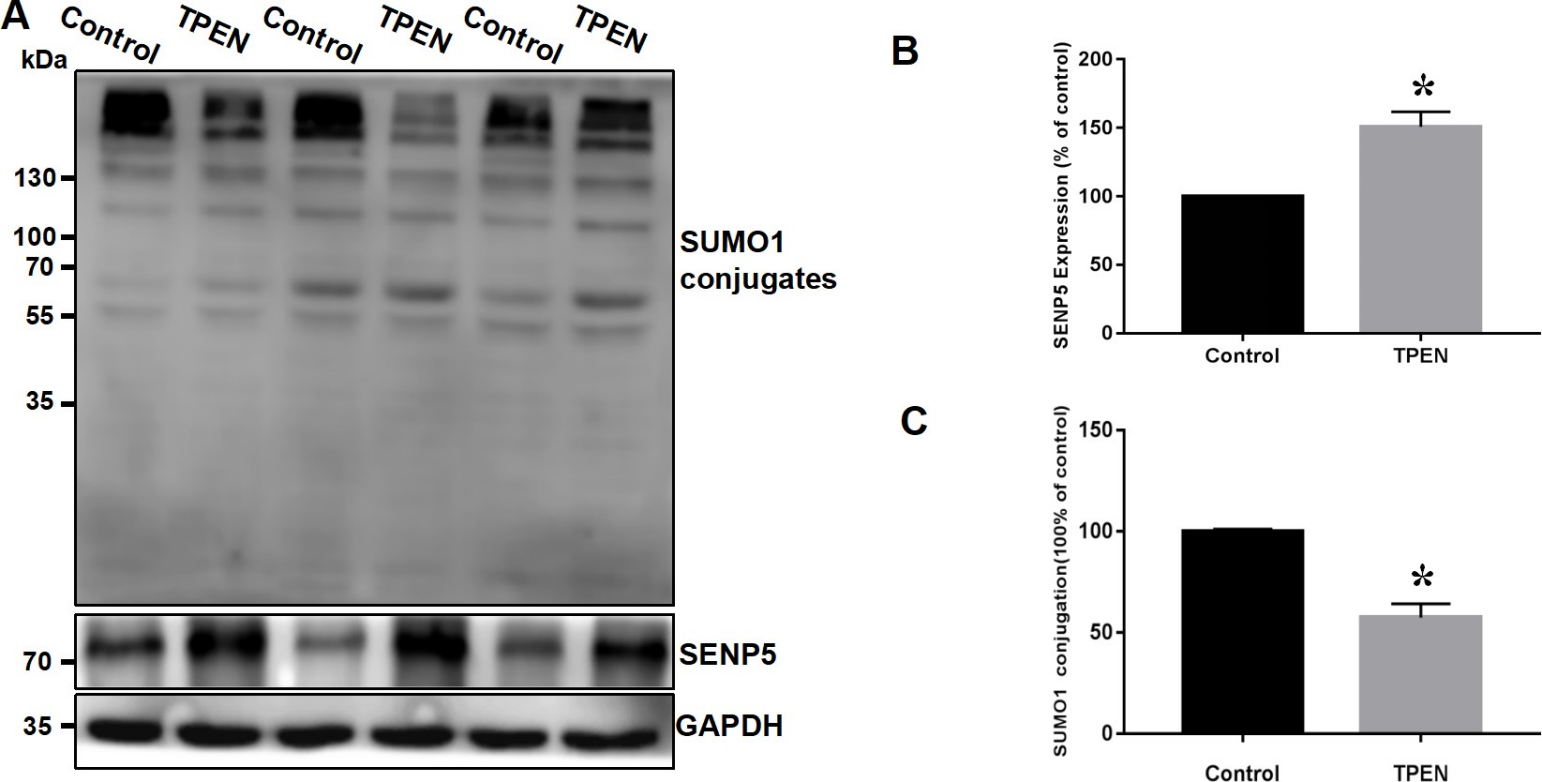
SUMOylated protein and SENP5 protein expression in zinc-deficient hiPSC-CMs. (A) Western blot showing SUMO1-conjugated protein and SENP5 expression in normal and zinc-deficient hiPSC-CMs (n = 6). (B) Summarized data of SENP5 protein expression in normal and zinc-deficient hiPSC-CMs. Values are presented as the mean ± SD Statistical significance was analyzed using Tukey’s test. *P < 0.05 vs. control. (C) Summarized data of SUMOylated proteins. Values are presented as the mean ± SD. Statistical significance was analyzed using Tukey’s test. *P < 0.05 vs. control.

**Fig 6:**
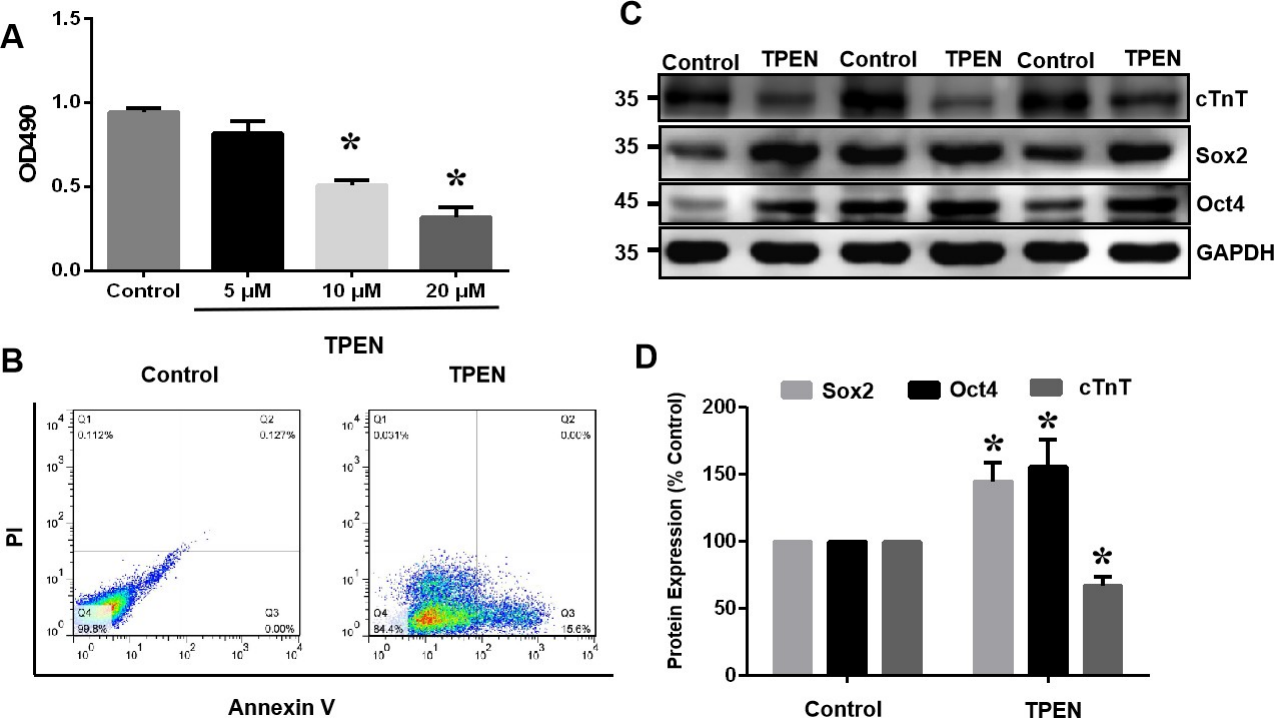
Zinc deficiency affects apoptosis, activity, and differentiation of hiPSC-CMs. (A) Cell viability. Compared with the control, TPEN (5, 10, and 20 μM) significantly decreased hiPSC-CM viability. Values are presented as the mean ± SD. Statistical significance was analyzed using Tukey’s test. *P < 0.05 vs. control (n = 6). (B) Flow cytometric analysis of the apoptotic state. Compared with the control, the selective zinc chelator TPEN (10 μM, 2 h) significantly increased apoptosis of hiPSC-CMs (n = 3). (C) Western blotting of Sox2, Oct4 and cTnT protein expression. In the zinc-deficient state, the expression levels of TnT were decreased in hiPSC-CMs, while the expression level of stem cell marker, Sox2 and Oct4 were increased (n = 6). (D) Summarized data of (C). Values are presented as the mean ± SD Statistical significance was analyzed using Tukey’s test. *P < 0.05 vs. control.

Human induced pluripotent stem cells (hiPSCs) were first established by Takahashi et al., who introduced a lentiviral expression vector carrying a set of transcription factors (Oct4, Sox2, Nanog, and LIN28) into adult human fibroblasts [28]. HhiPSC-CMs differentiated from hiPSCs was obtained, and were founded to be immunopositive for cardiomyocyte biomarkers such as cardiac troponin I (cTnI) and cardiac troponin T (cTnT). To assess the role of SENP5 in abnormal development of the myocardium caused by zinc deficiency *in vitro*, hiPSC-CMs with selective zinc chelator TPEN was evaluated and the SENP5 protein expression was examined by western blot analysis firstly. As shown in Fig 5, TPEN significantly induced an increase in SENP5 and decrease in the covalent binding of SUMO1 to target proteins in hiPSC-CMs (*P* < 0.05). Further to confirm the effects of zinc deficiency on cardiomyocytes, the effect of TPEN on the viability, apoptosis, and differentiation of hiPSCs and hiPSC-CMs was investigated. As shown in Fig 6A, after adding 5, 10, or 20 μM TPEN for 2 h, cell viability was decreased, especially decreasing by more than 40% at 10 μM (*P* < 0.05). Additionally, flow cytometry results showed that most hiPSC-CMs were in apoptotic state upon zinc deficiency (Fig 6B). Under zinc-deficient status, cTnT expression levels were significantly decreased, while the levels of the stem cells index, Sox2 and Oct4, were increased in hiPSC-CMs (*P* < 0.05; Fig 6C and 6D). These results indicated that zinc deficiency induced apoptotic cells, decreased cell viability, and inhibited the differentiation ability of hiPSCs into hiPSC-CMs.

### SENP5 knockdown antagonizes the effect of TPEN on apoptosis and differentiation of hiPSC-CMs

**Fig 7:**
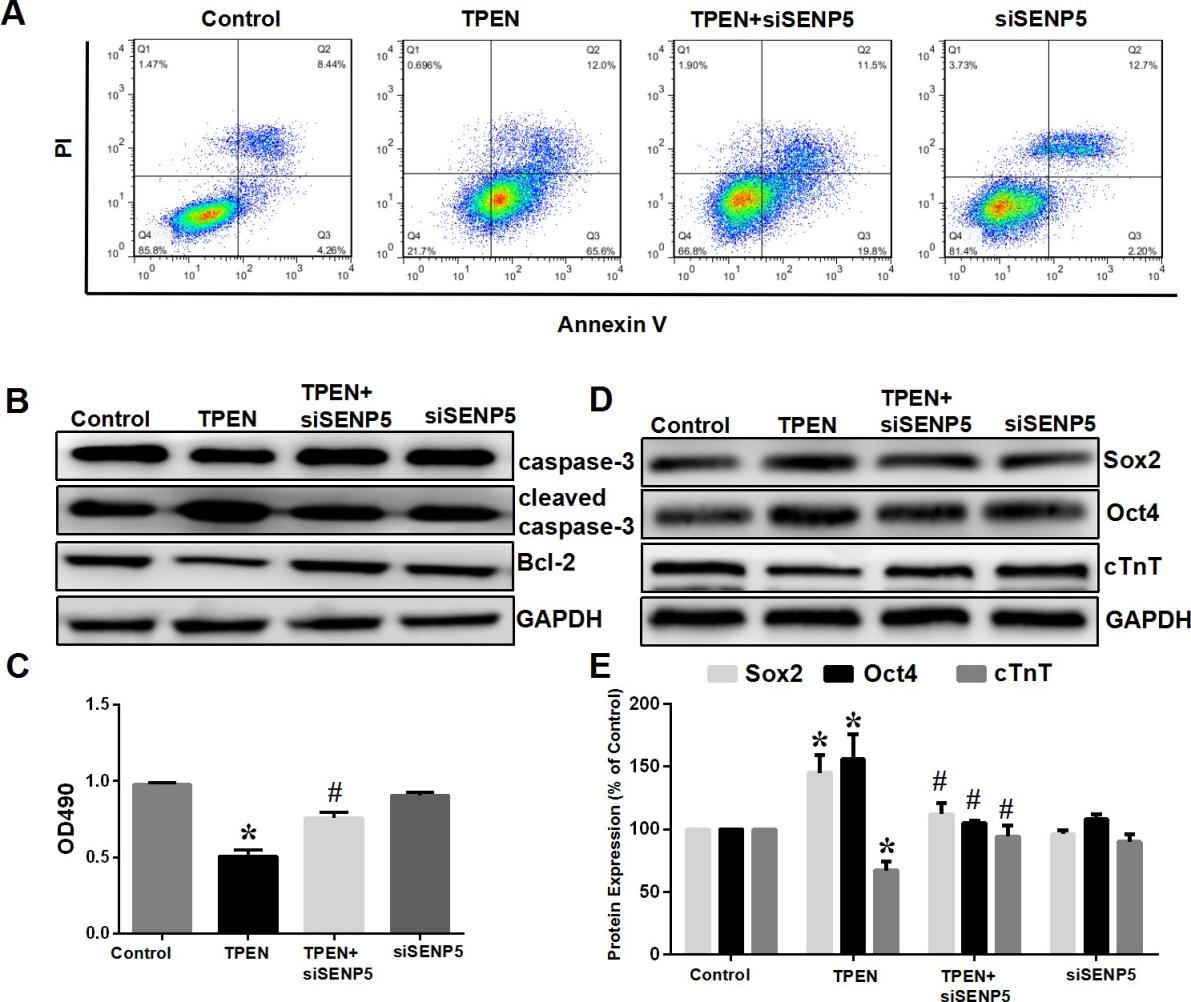
SENP5 knockdown antagonizes the effect of TPEN on apoptosis and induced abnormal differentiation of hiPSCs. (A) Flow cytometric analysis of the apoptotic state (n = 3). (B) Western blot analysis of caspase-3, active caspase-3 and Bcl-2 levels in cardiac progenitor cells. Viability of hiPSC-CMs with siSENP5 was increased slightly compared with zinc-deficient hiPSC-CMs (n = 6). (C) CCK8 assay of cell viability. TPEN (10 μM) significantly decreased hiPSC-CM viability, which was abrogated by siSENP5 (n = 6). *P < 0.05 vs. control; ^#^*P* < 0.05 vs. TPEN. (D) Western blotting of Sox2, Oct4 and cTnT, protein expression. In the zinc-deficient state, expression levels of cTnT were decreased in hiPSC-CMs, while the expression level of stem cell marker Sox2 and Oct4 were increased, which were abrogated by siSENP5. (E) Summarized data of (B). Values are presented as the mean ± SD. Statistical significance was analyzed by Tukey’s test. *P < 0.05 vs. control; ^#^*P* < 0.05 vs. TPEN.

According to the previous results, a zinc deficiency induces increased expression of SENP5. Using the SENP5 small interfering RNA (siSENP5) which inhibited SENP5 expression in hiPSC-CMs, the effect of SENP5 on abnormalities caused by an absence of zinc *in vitro* was investigated further. Western blot analysis demonstrated that TPEN induced an increase in cleaved caspase-3 level, and caspase-3 and Bcl-2 proteins were decreased, while in the TPEN+siSENP5 medium, opposite effects were observed with deceased cleaved caspase-3 and increased levels of caspase-3 and Bcl-2. Combined with flow cytometry results, the absence of zinc increased apoptosis and decreased the viability of hiPSC-CMs, which was restored by siSENP5 (*P* <0.05; Fig 7A–7C). Similarly, zinc deficiency caused the differentiation index proteins, Sox2 and Oct4, to increase and the expression level of cTnT to decrease. The levels of Sox2, Oct4 and cTnT showed no changes with the deletion of SENP5 (P <0.05; Fig 7D and 7E). These results further suggest that SENP5 overexpression plays an important role in zinc deficiency inducing abnormal differentiation of cardiomyocytes and cardiac dysplasia.

## Discussion

During mouse embryogenesis, early cardiac morphogenesis can be divided into four main stages which is similar to human heart development. Early cardiac morphogenesis in mice can be divided into four major stages: the cardiac crescent period (E7.5), linear heart tube period (E8.0), looping heart period (E9.0), and chamber formation period (E10.5). After E10.5, the heart enters the stage of rapid cell proliferation, migration, and differentiation, thereby promoting ventricular and trabecular structures. Simultaneously, the core and chamber septum begin to form and divide the core into four chambers [29]. Therefore, fetal mice from E10.5 to E19.5 stages can be used to investigate the association between the association of SUMOylation and heart development.

The development of the heart is a complex process regulated by multiple genes and signaling pathways. The importance of SUMOylation is also underlined by the observation of cardiac abnormalities. The knockdown of SUMO1 in mice leads to atrial and ventricular septal defects [30]. SUMO-1 can prevent the negative effects of SERCA2a activity caused by the hypertrophied and failing myocardium [31]. The studied confirmed SUMO1 impact heart development. In this study, free SUMO1 and conjugated SUMO1 were founded to be differentially expressed during the heart development of fetal and neonatal mice. The expression of conjugated SUMO1 was gradually decreased though the entire cardiac developmental process, while the level of free SUMO1 was increased. It was also confirmed that the expression level of SENP5 protein was decreased during the heart development. This indicates that SENP5 could potentially be related to SUMO1 deconjugation to maintain the dynamic balance of SUMOylation and deSUMOylation, and play an important part in heart development. Previous studies revealed that the ability of myocardium differentiation gradually diminishes following the developmental process. It indicated that SENP5 may be associated with the myocardial viability and differentiation. Further research will be carried out in the following experiments.

Zinc is essential for normal growth and development of embryos and fetuses [32–36]. Pregnant rats with severe zinc deficiency can lead to abortion, stillbirth or fetal heart malformation [8], which suggests that zinc plays a role in the regulation of myocardial development. Compared with normal mice, the lack of zinc induced delayed development of heart, and the thicknesses of both the ventricular wall and interventricular septum were decreased. HiPSCs are widely used in various areas of cardiovascular research, and are some efficient methodologies for human induced pluripotent stem cells (hiPSCs) cardiac differentiation to elucidate mechanisms of cardiomyogenesis [37]. This was investigated by culturing hiPSCs to explore the effect of zinc deficiency on viability and differentiation of cardiomyocytes. We demonstrated that a zinc deficiency significantly increased the number of apoptotic hiPSC-CMs and the expression level of Sox2 and Oct4 protein, while decreasing the protein expression levels of cTnT in hiPSC-CMs. These results suggest zinc deficiency induces abnormal differentiation of hiPSCs and apoptosis of hiPSC-CMs. While the amount of zinc is clearly important and under dynamic temporal control, it is not well understood how zinc affects myocardial development.

There are several mechanisms by which zinc regulates myocardial development. Zinc deficiency during pregnancy leads to oxidative stress in the body, which changes the death pattern of cardiac neural crest blast cells that should develop into the heart. The number of cells is reduced, which leads to abnormal development of the embryonic heart. Studies have shown that SUMOylation antagonizes oxidative stress [38], while the SENP family undergoes changes in subcellular distribution during oxidative stress [39]. Our study found that zinc-deficient status induced a decreased level of SUMO1 and a increasing level of SENP5. The effect of zinc deficiency, and relationship to SENP5, was investigated further. SENP5 was founded to be overexpressed in a zinc-deficient state, and de-SUMOylation was enhanced, which led to abnormal hiPSC differentiation and increased apoptosis of hiPSC-CMs. It is speculated that SENP5 may have an essential role in the effect of zinc. To further detect the concrete action of SENP5 in zinc deficiency, SENP5-deficient cells were established through treatment with siSENP5. Although cells were in the absence of zinc, the abnormal differentiation of hiPSCs and apoptosis of the hiPSC-CMs, under the suppression of SENP5, was not found. This suggested that SENP5 overexpression, potentiated by zinc deficiency, induces abnormal development of myocardial cells.

In this study, the partial mechanism of cardiac dysplasia caused by zinc deficiency was elucidated. SENP5 regulates the SUMO1 deconjugation during heart development. Zinc deficiency induced abnormal hiPSC differentiation, increased cells apoptosis, and decreased the viability of hiPSC-CMs by inducing SENP5 overexpression, which caused myocardial abnormality. Therefore, this study may provide a new strategy for the prevention and treatment of clinical CHD.

## Supporting information

S1 Raw images (PDF)
