# Supplementary figures and images for: Zinc deficiency induces abnormal development of the myocardium by promoting SENP5 overexpression
Source: PLoS One. 2020 Nov 19;15(11):e0242606. doi: 10.1371/journal.pone.0242606 (PMC7676719; doi:10.1371/journal.pone.0242606)

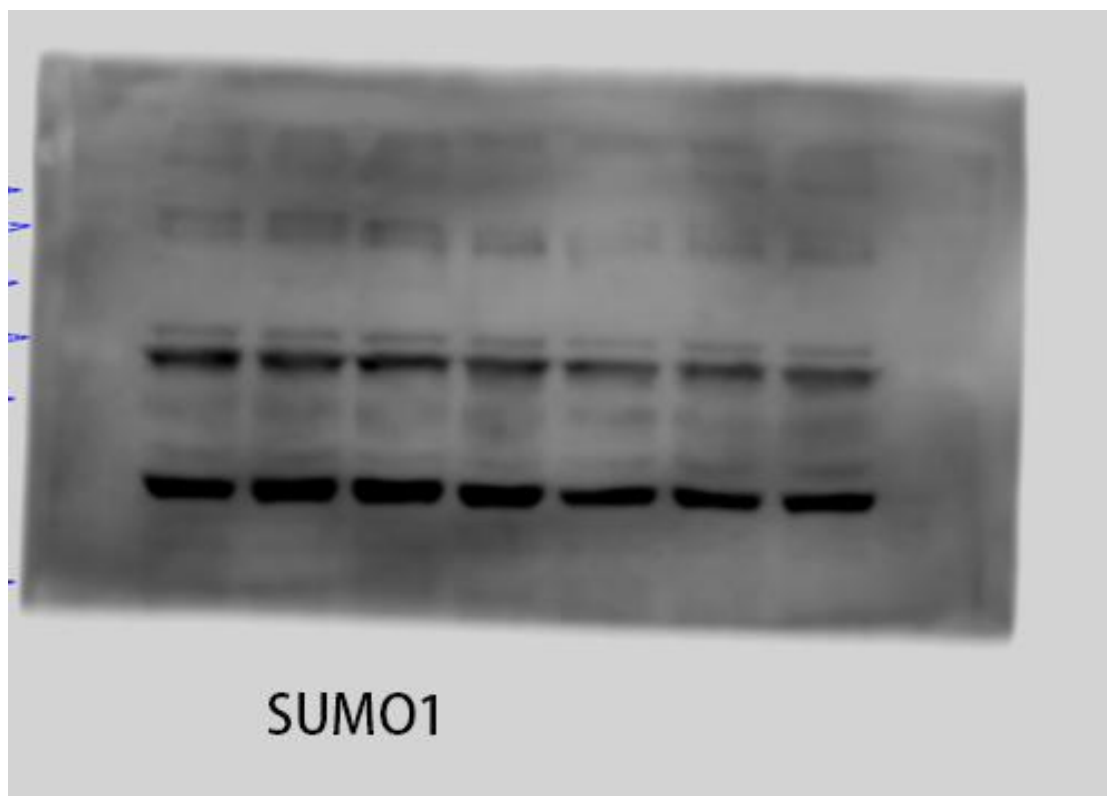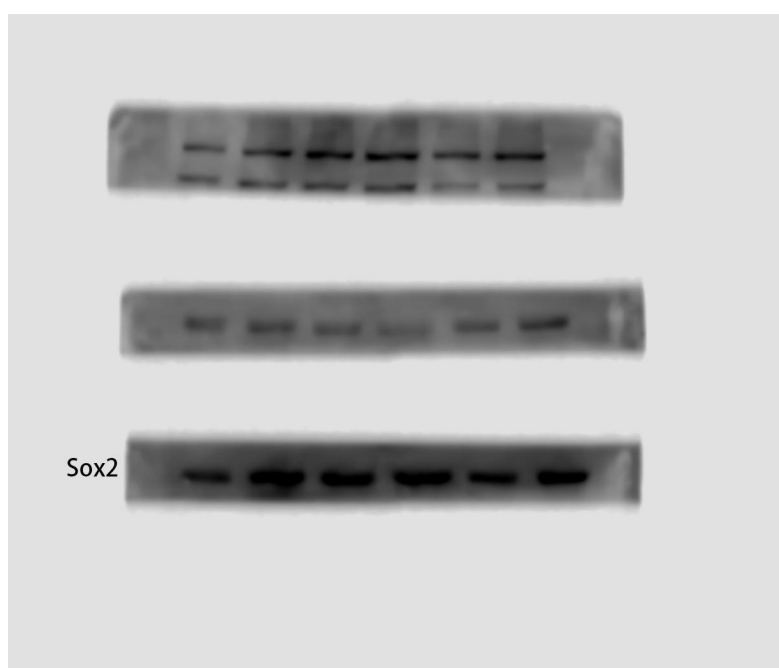

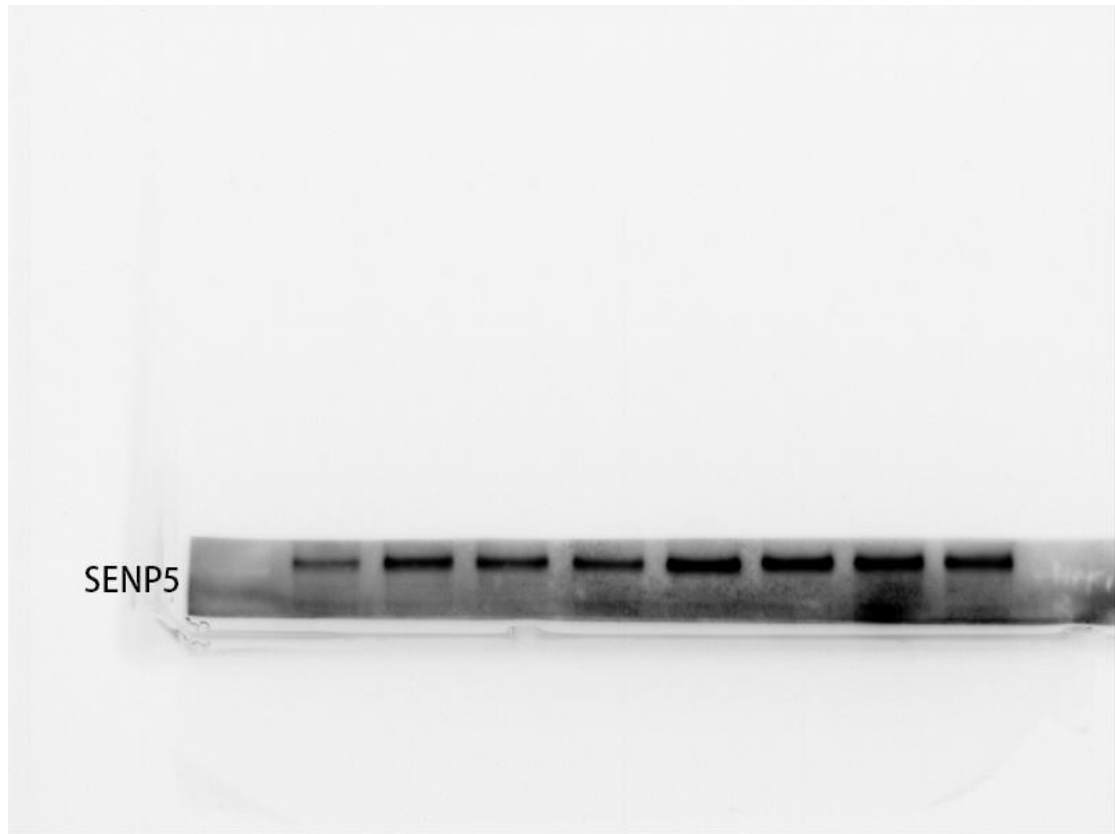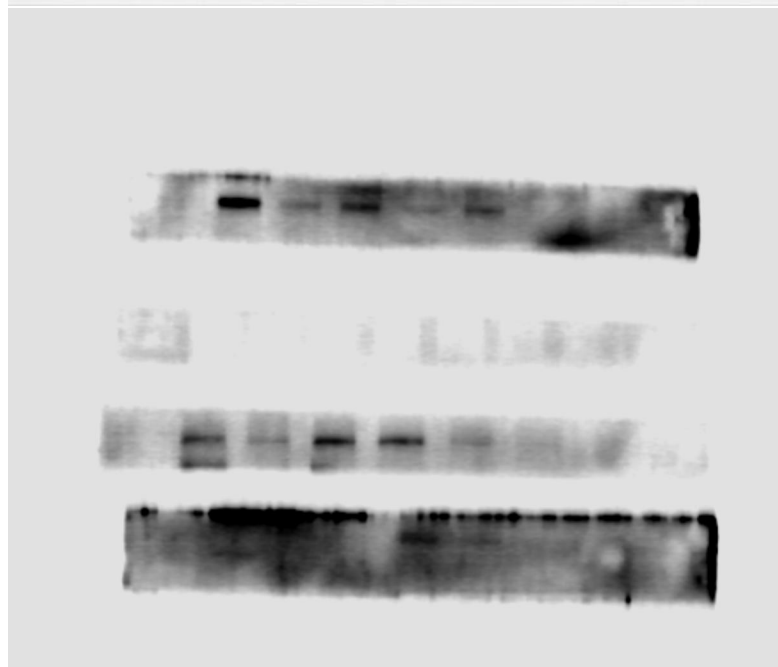

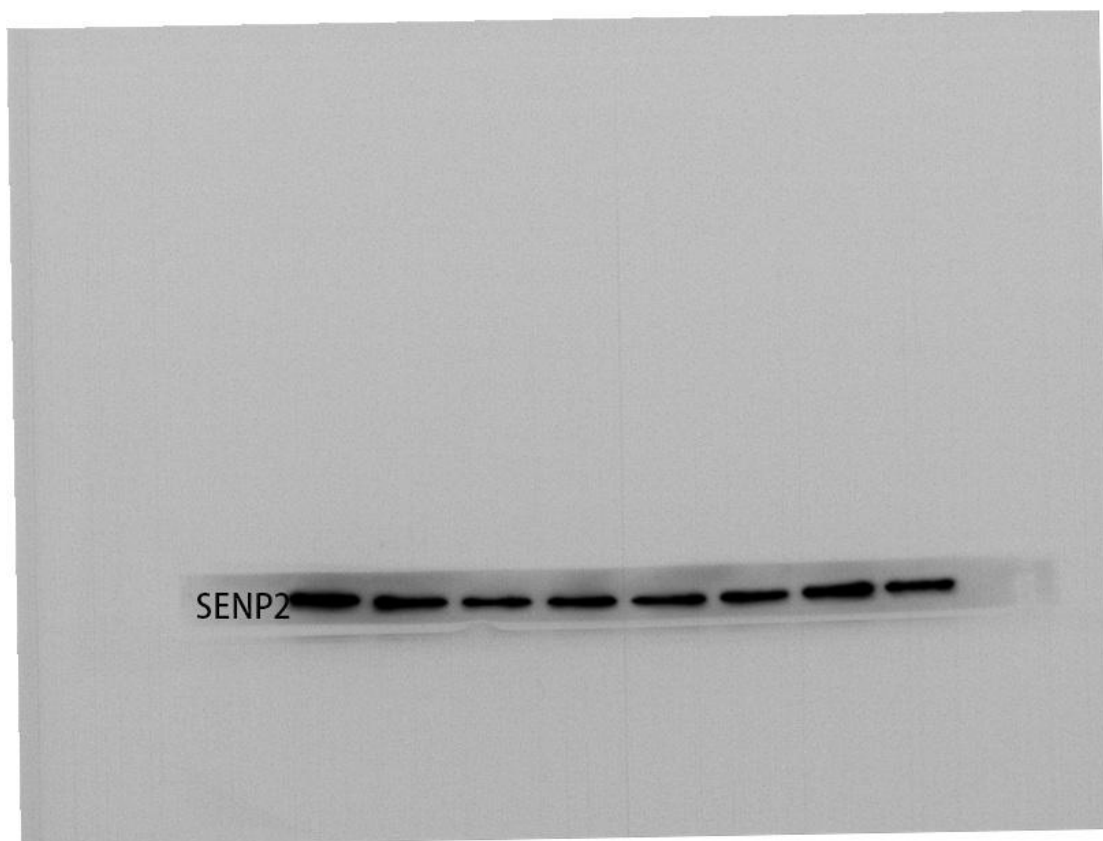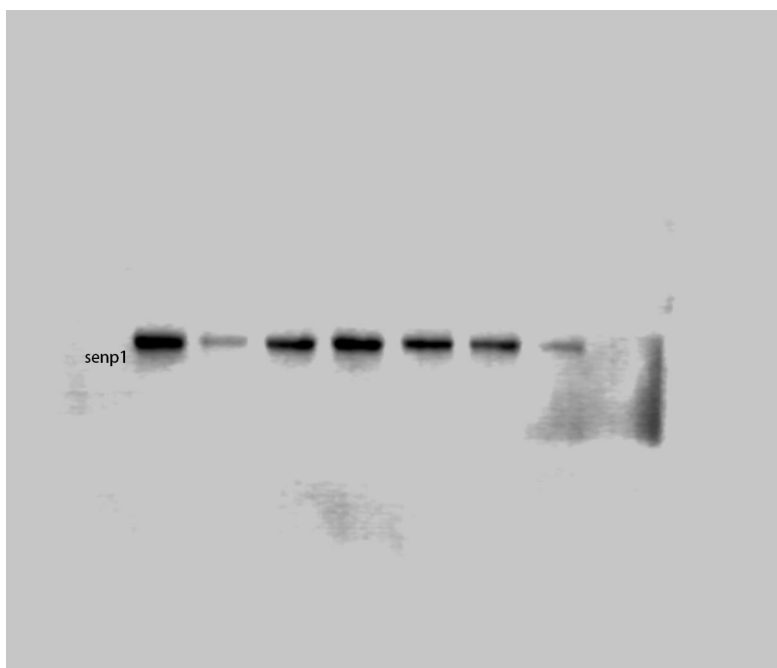

GAPDH

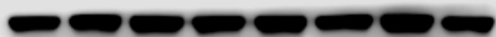

GAPDH

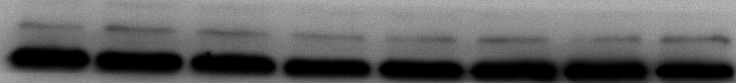

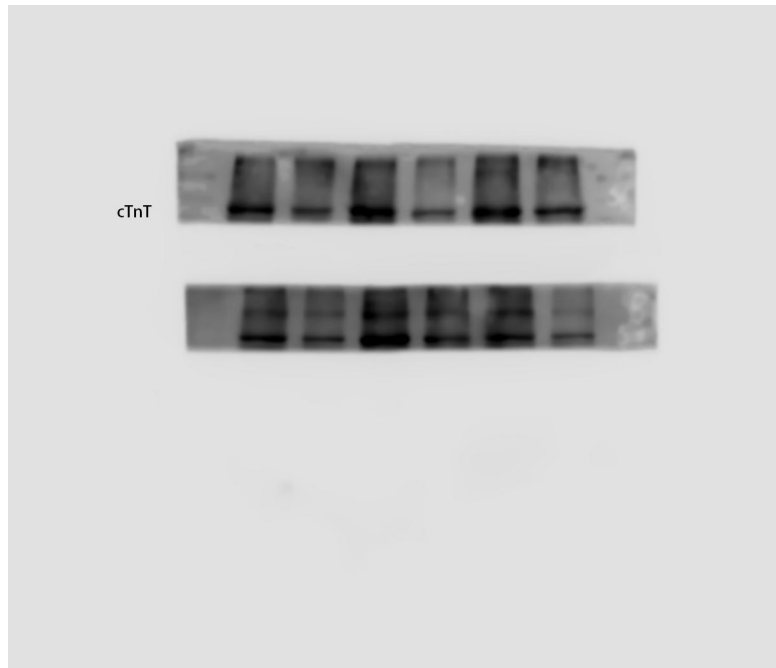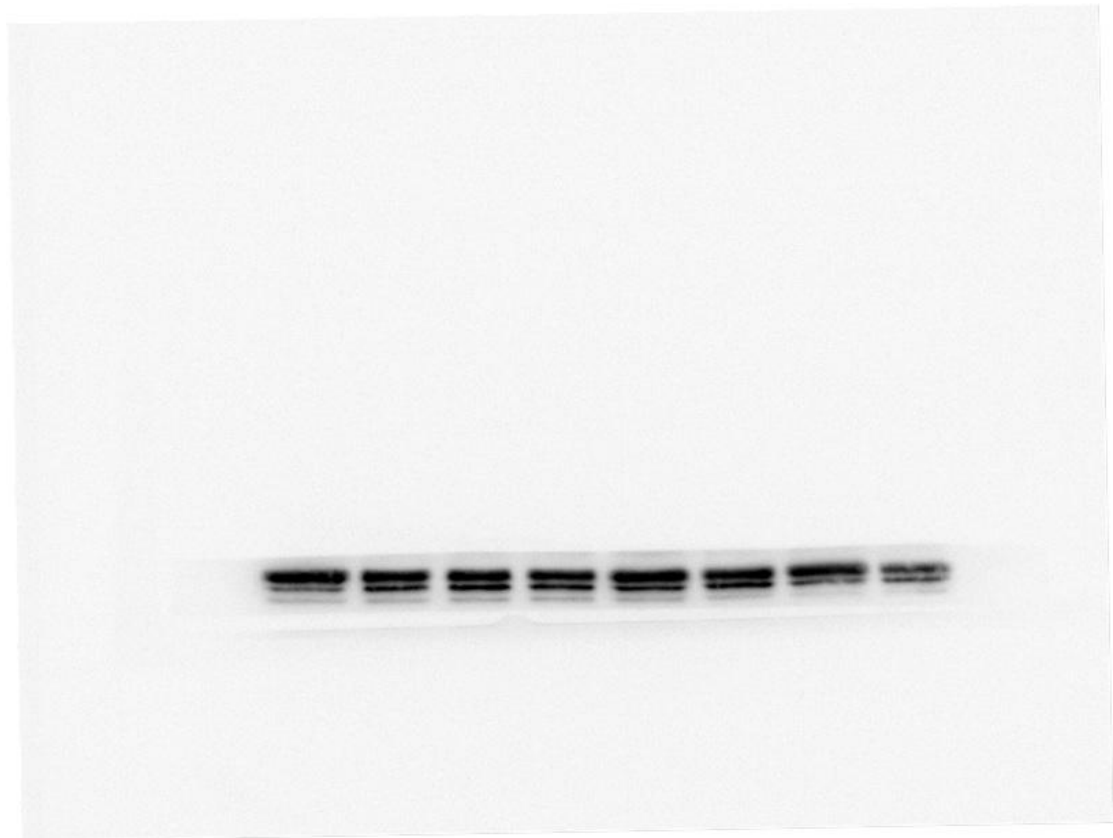

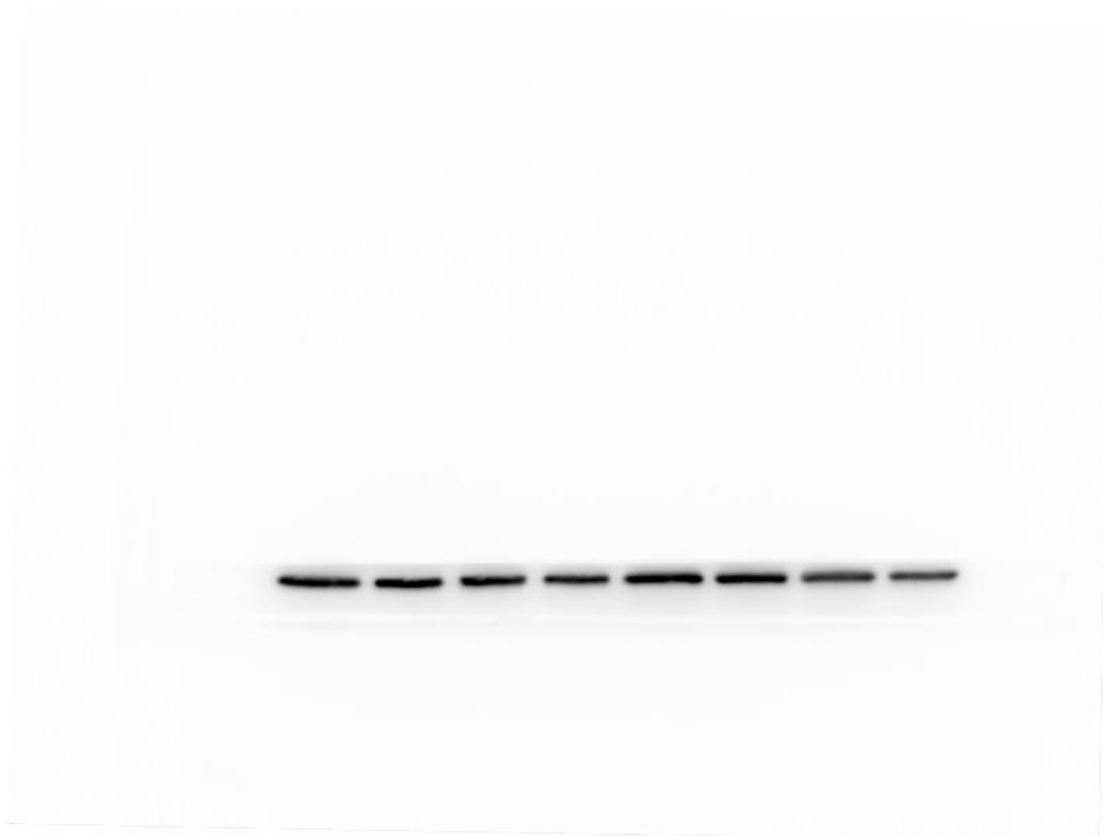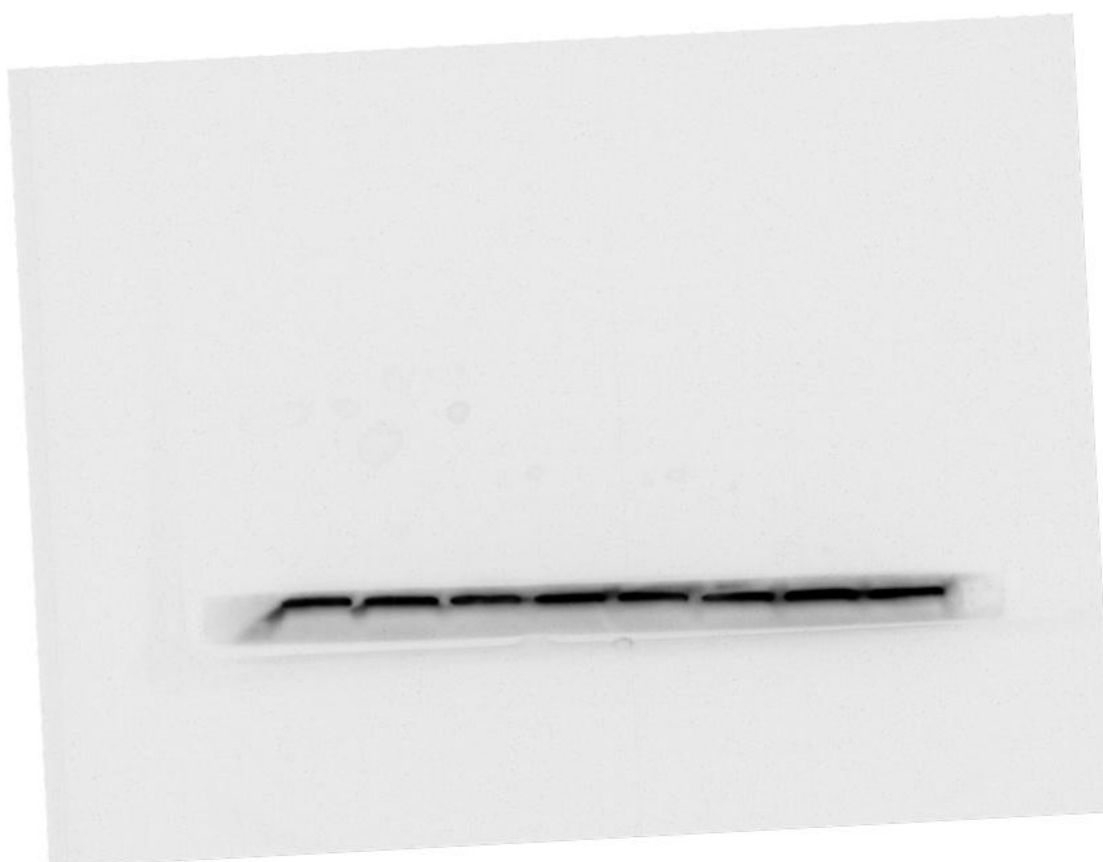

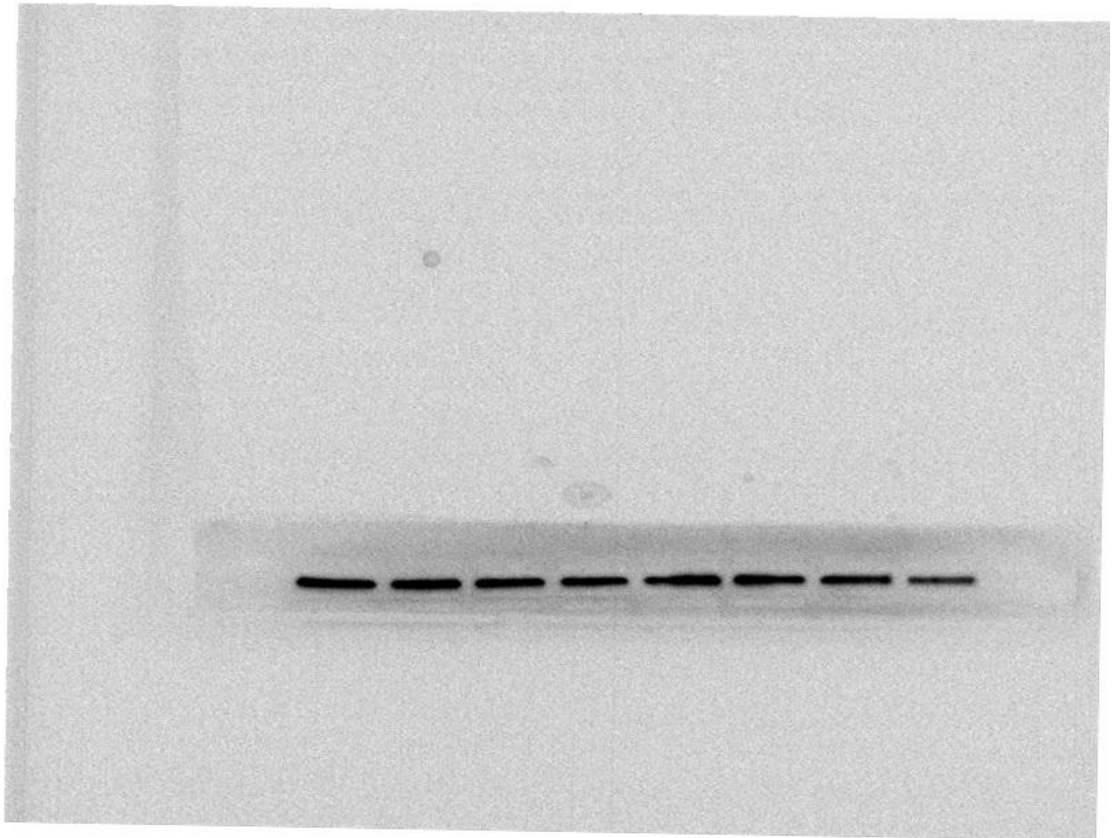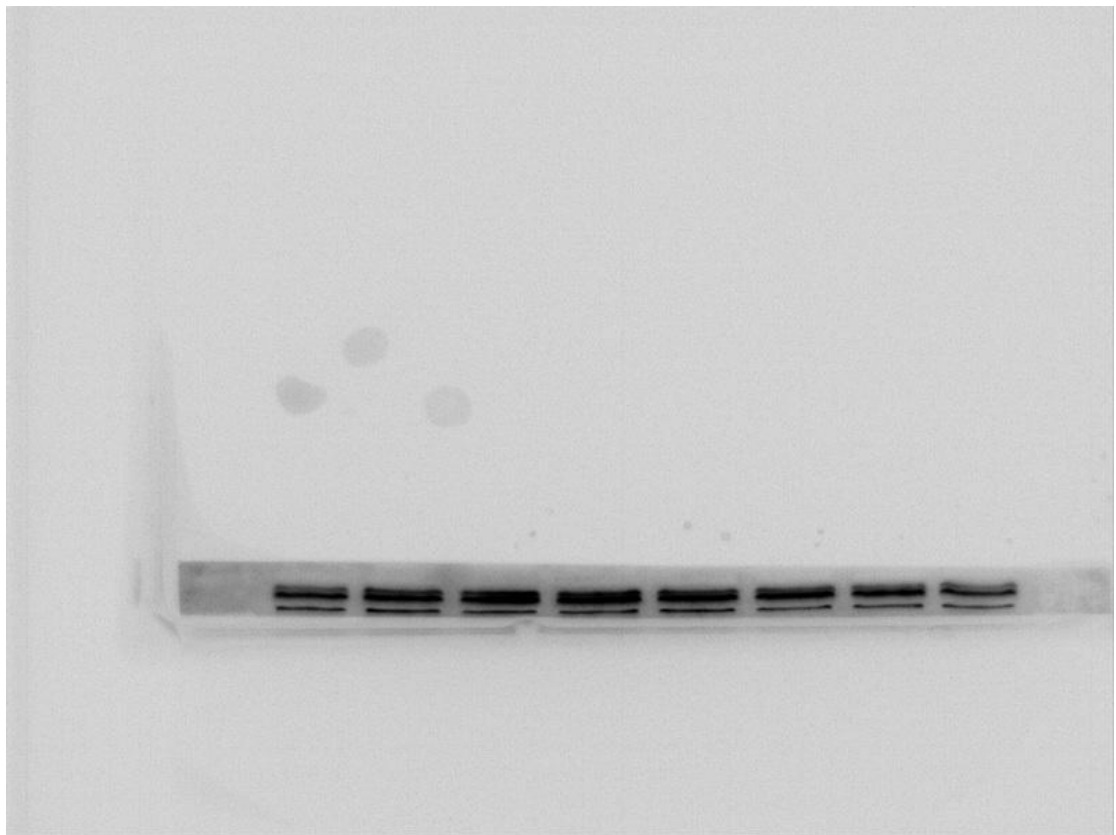

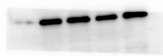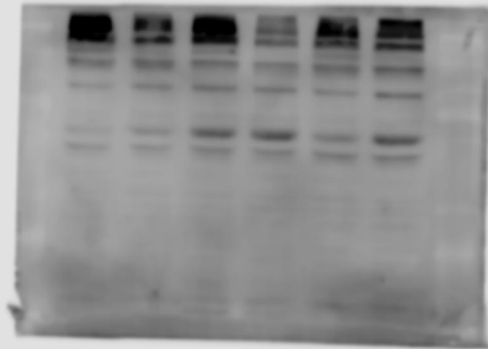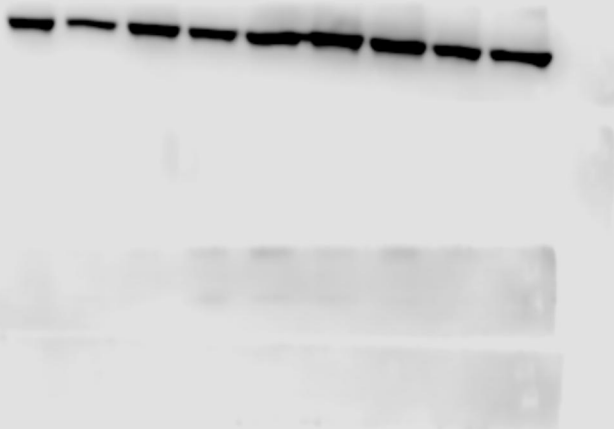

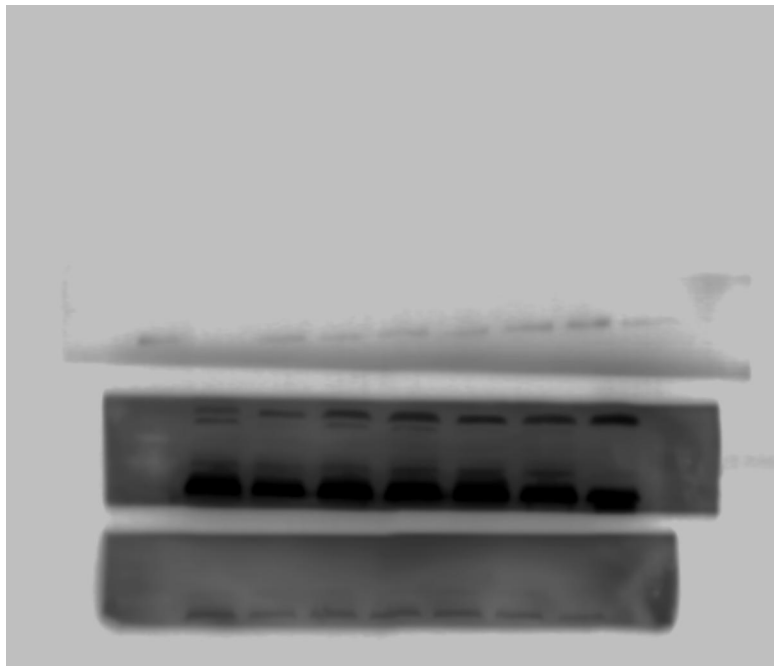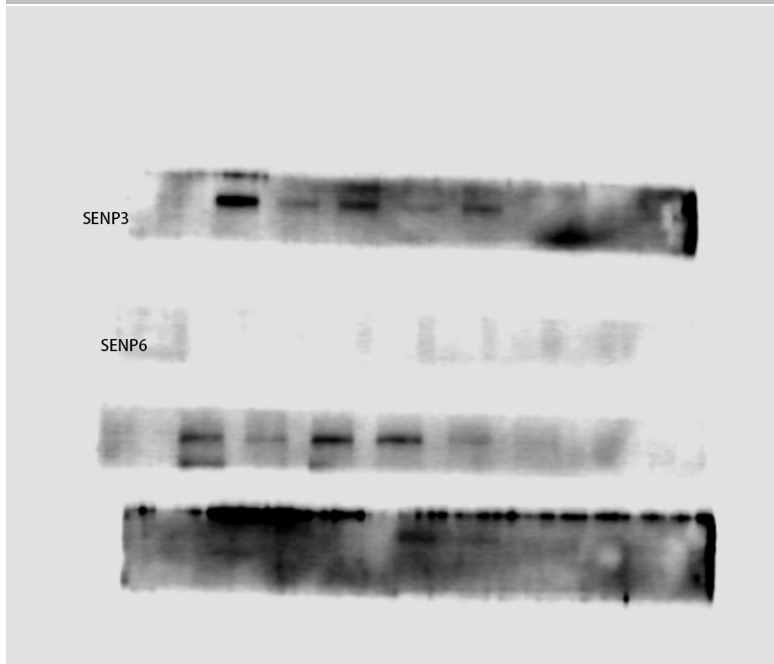

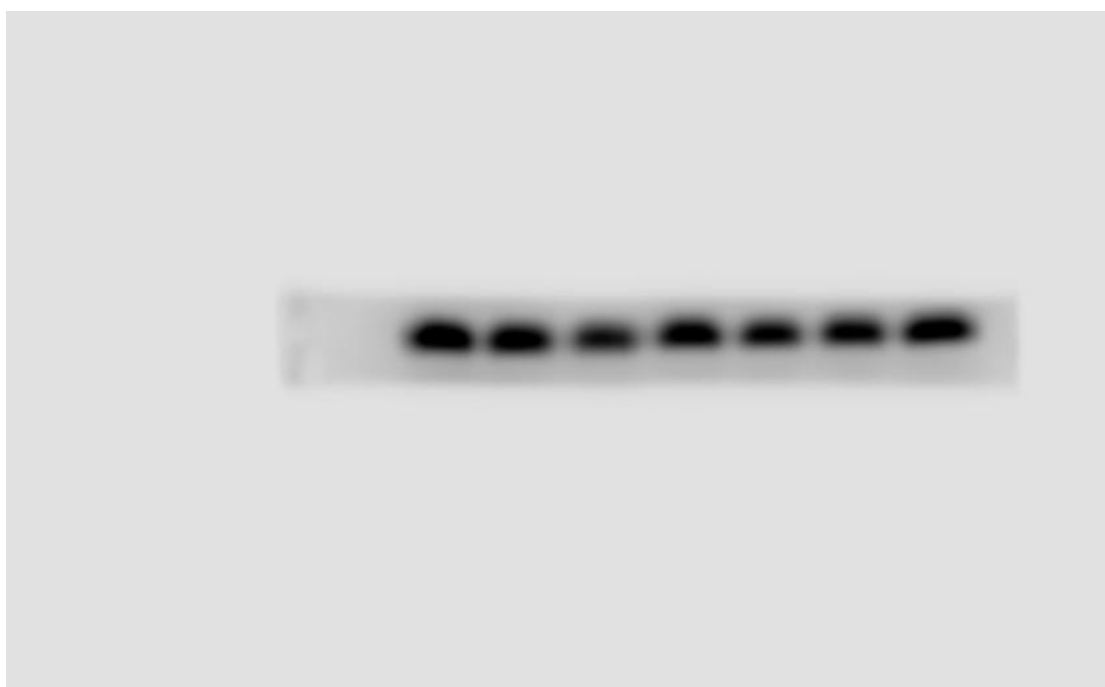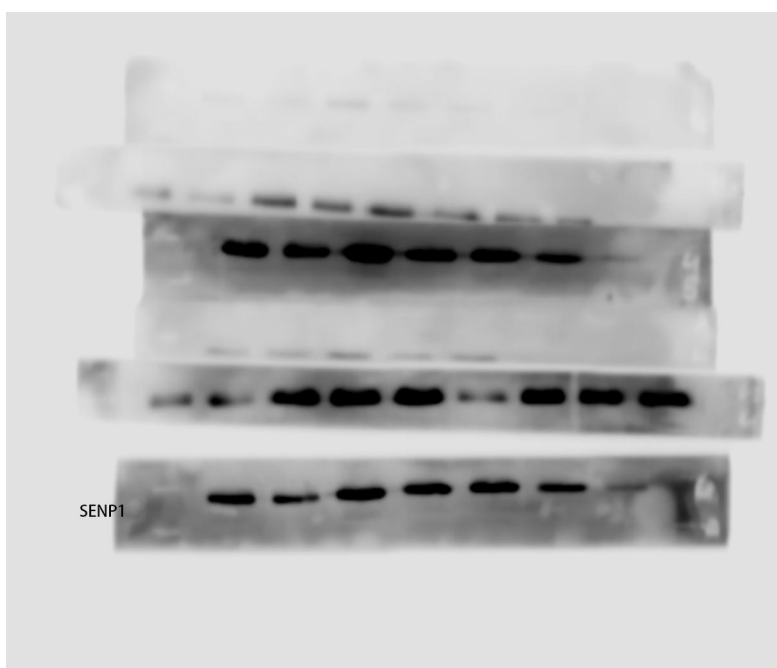

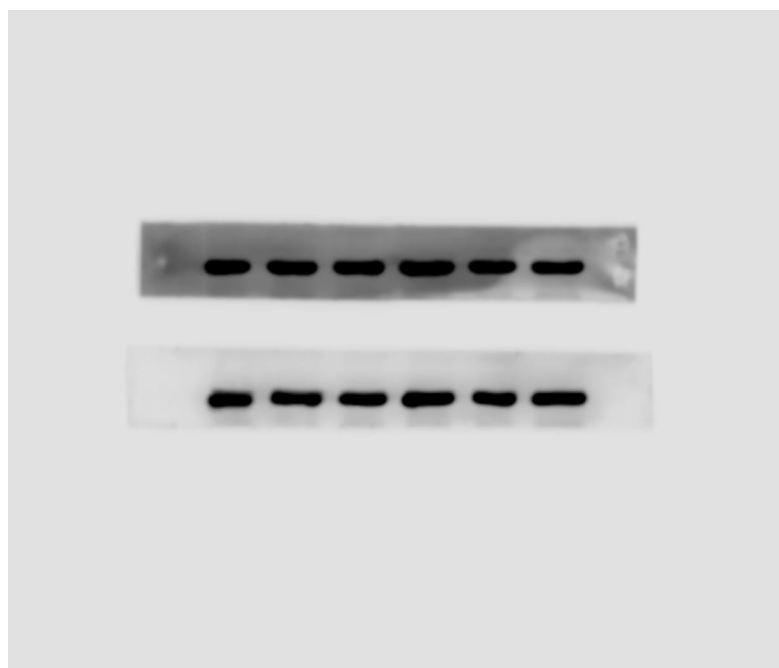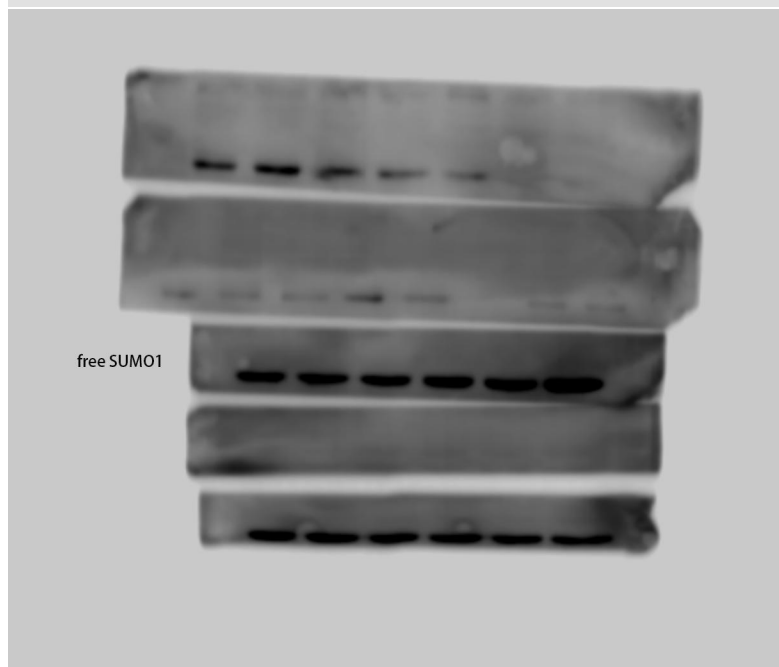

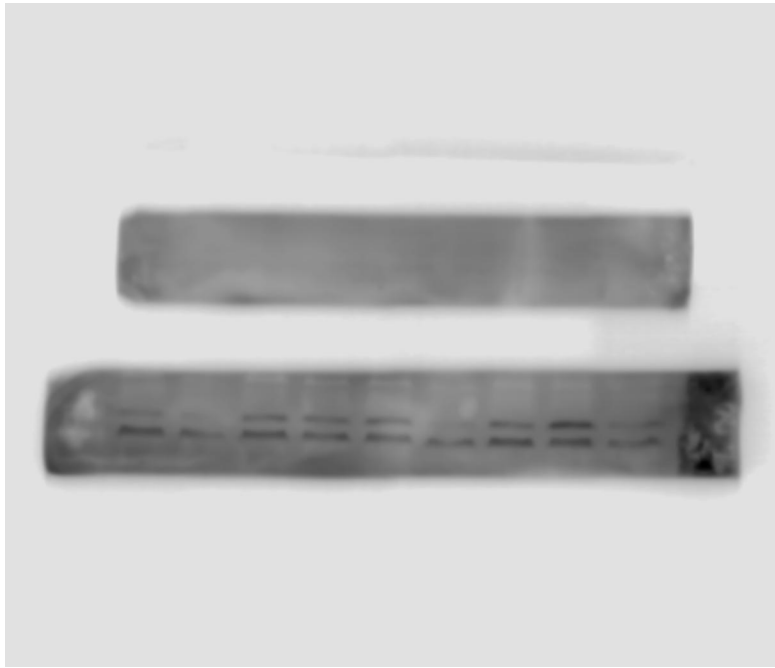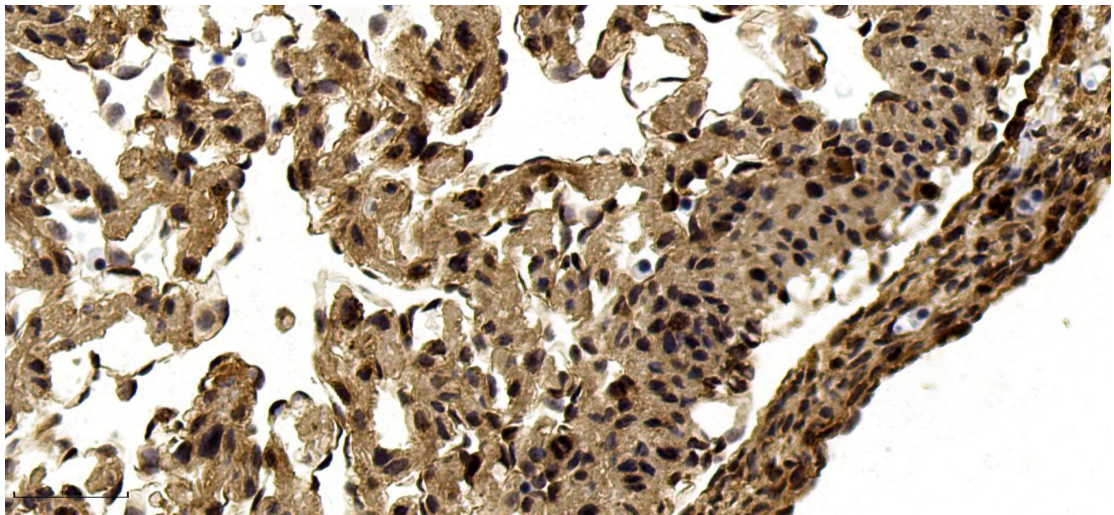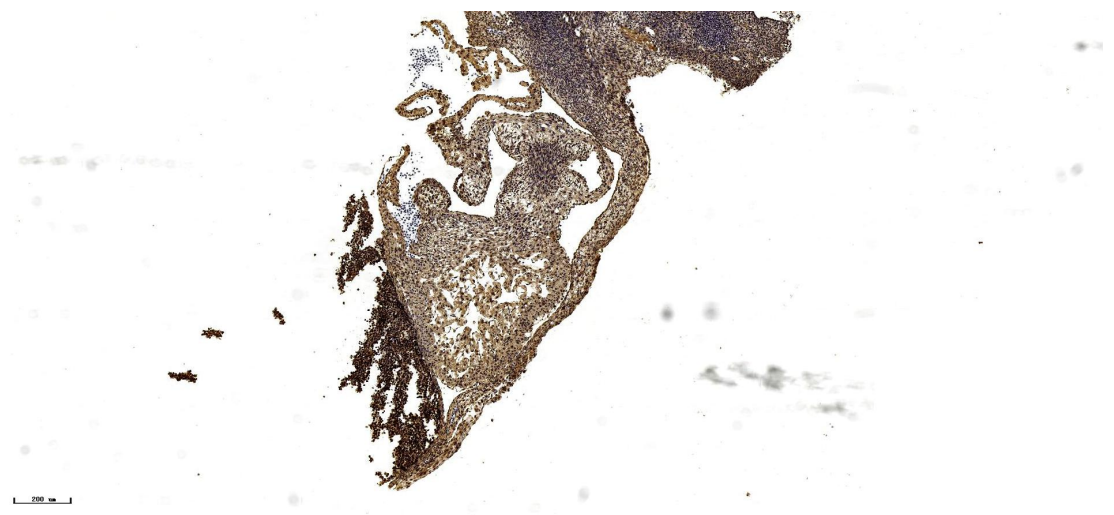

Supplement: S1 Raw images — (PDF) [file pone.0242606.s001.pdf]
